# Draft genome sequence of *Methyloversatilis* sp. strain NSM2, isolated from a wastewater treatment plant

**DOI:** 10.1128/mra.00952-24

**Published:** 2024-10-22

**Authors:** Alicia Aranda, David Primo-Catalunya, Maite Pijuan, José L. Balcázar

**Affiliations:** 1 Catalan Institute for Water Research (ICRA-CERCA), Girona, Spain; Department of Biology, Queens College, Queens, New York, USA

**Keywords:** *Methyloversatilis *sp., methane, wastewater

## Abstract

Here, we report the draft genome sequence of *Methyloversatilis* sp. NSM2, isolated from a wastewater treatment plant. This strain was enriched using methane as the sole carbon source. The 4.5-Mb genome, with a GC content of 66.5%, provides valuable insights into the strain’s ecological role and metabolic capabilities.

## ANNOUNCEMENT

The genus *Methyloversatilis* comprises Gram-negative, non-motile, rod-shaped, non-spore-forming, and facultatively methylotrophic bacteria that can grow on single-carbon compounds such as methanol (1–3). Previous studies have shown that this genus dominates among methylotrophic bacteria in sequencing batch denitrifying reactors (4–6). Due to their metabolic versatility, *Methyloversatilis* species play significant roles in the carbon cycle, particularly in the transformation of single-carbon compounds in different ecosystems (7).

Currently, the *Methyloversatilis* genus comprises three species that have been validly published and hold standing in nomenclature (https://lpsn.dsmz.de/genus/methyloversatilis) (8). Here, we present the draft genome sequence of *Methyloversatilis* sp. strain NSM2, which is closely related to the type strain of *Methyloversatilis discipulorum* FAM1^T^ (GenBank accession number: NZ_AZUP01000001.1).

Strain NSM2 was isolated from the aerobic bioreactor at the municipal wastewater treatment plant in Girona. Mixed liquor sludge was used as the inoculum to enrich methanotrophic bacteria in 0.5-L gas-tight bottles containing 100 mL of modified NMS medium (mod-NMS) (9), with the following composition (mg/L): NaNO_3_, 3,400; MgSO_4_·7H_2_O, 1,000; CaCl_2_·2H_2_O, 298; KH_2_PO_4_, 272; K_2_HPO_4_, 610; Ferric EDTA, 4.1 mL/L; N-allylthiourea, 20; and trace elements solution (1 mL/L) with Na_2_-EDTA·2H_2_O, 451.6; ZnSO_4_·7H_2_O, 10; MnCl_2_·4H_2_O, 3; H_3_BO_3_, 30; Na_2_MoO_4_·2H_2_O, 3; FeSO_4_·7H_2_O, 200; NiCl_2_·6H_2_O, 2; and CoCl_2_·6H_2_O, 20. Methane (20%, vol/vol) was used as the sole carbon source, with incubation at 28°C and 150 rpm.

After a 10-month enrichment period with several transfers to fresh mod-NMS, 100 µL of serial dilutions of the mixed culture was spread on mod-NMS agar plates and incubated in a methane-enriched atmosphere at 28°C. Subsequently, single colonies were isolated and subcultured on the same medium. Genomic DNA was extracted using a standard phenol-chloroform protocol, and the sequencing DNA library was prepared with the Nextera XT DNA Library Prep Kit (Illumina, San Diego, CA, USA) following the manufacturer’s instructions. Paired-end sequencing was conducted using the Illumina NovaSeq 6000 system at Macrogen Inc. (Seoul, Republic of Korea), with an average read length of 151 bp.

The reads were filtered using Cutadapt 3.5 (10), applying a Q-score threshold of >20 and a minimum length of >100 bp. Read quality was assessed with FastQC version 0.11.9 (11). Genome assembly was performed using SPAdes version 3.15.5 (12), and its quality was assessed using QUAST version 5.0.2 (13). Contigs with coverage greater than 150× were selected and reordered using progressiveMauve software (Mauve version 2.4.0) (14), with the genome of *Methyloversatilis discipulorum* FAM1^T^ as the reference. Draft genome annotation was performed with the NCBI Prokaryotic Genome Annotation Pipeline (15).

The final assembled genome had a total length of 4.5 Mb, with a GC content of 66.5% and 170× coverage. The assembly consisted of 19 contigs, with an *N*50 value of 658.1 kb. Completeness and contamination were assessed using CheckM version 1.2.2 (16), indicating 98.73% completeness and 1.93% contamination. The *Methyloversatilis* sp. NSM2 genome comprises 4,132 protein-coding genes, 14 pseudogenes, and 53 RNA-coding genes, including 46 tRNAs, 3 rRNAs (5S, 16S, and 23S), and 4 non-coding RNAs. Overall, this study shows that *Methyloversatilis* sp. NSM2 lacks known methane monooxygenase genes and may use an unidentified alternative pathway for methane metabolism, warranting further investigation.

## Data Availability

This Whole Genome Shotgun project has been deposited in NCBI GenBank under BioProject accession number PRJNA1090027, BioSample accession number SAMN40554059, and GenBank accession number JBBUKV000000000. The version described in this paper is the first version, JBBUKV000000000.1. The raw sequencing reads have been deposited in the NCBI Sequence Read Archive under accession number SRR30615129.
